# Endothelial Cdc42 deficiency impairs endothelial regeneration and vascular repair after inflammatory vascular injury

**DOI:** 10.1186/s12931-018-0729-8

**Published:** 2018-02-08

**Authors:** Jiawen Lv, Junchao Zeng, Fukun Guo, Yiran Li, Mengying Xu, Yuanxiong Cheng, Lin Zhang, Shaoxi Cai, Yinghua Chen, Yi Zheng, Guodong Hu

**Affiliations:** 1 Department of Respiratory and Critical Care Medicine, Nanfang Hospital, Southern Medical University, Guangzhou, 510515 China; 2 Guangdong Provincial Key Laboratory of Construction and Detection in Tissue Engineering, Department of Histology and Embryology, School of Basic Medical Sciences, Southern Medical University, Guangdong Provincial Key Laboratory of Construction and Detection in Tissue Engineering, Guangzhou, 510515 China; 3 Division of Experimental Hematology and Cancer Biology, Children’s Hospital Research Foundation, 3333Burnet Avenue, Cincinnati, OH 45229 USA; 4 Division of Experimental Hematology and Cancer Biology, Cincinnati Children’s Hospital Research Foundation, University of Cincinnati College of Medicine, Cincinnati, OH USA

**Keywords:** ALI/ARDS, Pulmonary microvascular injury and repair, Cre/Loxp technique, Cdc42, PAK1/AKT pathway

## Abstract

**Background:**

Endothelial cell (EC) regeneration is essential for inflammation resolution and vascular integrity recovery after inflammatory vascular injury. Cdc42 is a central regulator of cell survival and vessel formation in EC development. However, it is unknown that whether Cdc42 could be a regulating role of EC repair following the inflammatory injury in the lung. The study sought to test the hypothesis that Cdc42 is required for endothelial regeneration and vascular integrity recovery after LPS-induced inflammatory injury.

**Methods and results:**

The role of Cdc42 for the regulation of pulmonary vascular endothelial repair was tested in vitro and in vivo. In LPS-induced acute lung injury (ALI) mouse models, knockout of the Cdc42 gene in ECs increased inflammatory cell infiltration and pulmonary vascular leakage and inhibited vascular EC proliferation, which eventually resulted in more severe inflammatory lung injury. In addition, siRNA-mediated knockdown of Cdc42 protein on ECs disrupted cell proliferation and migration and tube formation, which are necessary processes for recovery after inflammatory vascular injury, resulting in inflammatory vascular injury recovery defects.

**Conclusion:**

We found that Cdc42 deficiency impairs EC function and regeneration, which are crucial in the post-inflammatory vascular injury repair process. These findings indicate that Cdc42 is a potential target for novel treatments designed to facilitate endothelial regeneration and vascular repair in inflammatory pulmonary vascular diseases, such as ALI/ARDS.

**Electronic supplementary material:**

The online version of this article (10.1186/s12931-018-0729-8) contains supplementary material, which is available to authorized users.

## Background

Endothelial cell (EC) regeneration is essential for inflammation resolution and vascular integrity recovery after inflammatory injury in the pulmonary vascular system [1, 2]. The vascular endothelium functions as a barrier that protects the vasculature from disruptive stimuli, maintains homeostasis and tissue-fluid balance and is an active organ system with the capacity to regenerate [3–5]. Defects in endothelial regeneration inhibit the vascular repair process, leading to suboptimal resolution of many phenomena related to inflammation, such as vascular hyperpermeability, neutrophil infiltration, and lung edema [6, 7].

Acute lung injury (ALI) and its most severe state, acute respiratory distress syndrome (ARDS), are critical clinical diseases with high annual mortality rates for which no specific pharmacologic therapies are available [8–10]. These diseases have multiple causes, including infection, toxin inhalation or physical injury. Sepsis is the main cause of ARDS in the clinical setting and results in a poor prognosis [9]. Components of cell wall of Gram-negative bacteria such as lipopolysaccharide (LPS) released during sepsis will induce the recruitment of neutrophils to the lungs and secretion of pro-inflammatory cytokines. These serious inflammatory responses could cause the injury of ECs and disrupt the vascular integrity, leading to the lung edema [11, 12]. Therefore, lipopolysaccharide (LPS) has been widely used to generate ALI models via intratracheal administration [13, 14]. The pathogenesis of ALI is characterized by vascular damage causing extravasation of neutrophils around the vasculature and accumulation of protein-rich fluid in the alveolar cavity [15–17]. Vascular ECs have the capacity to rescue themselves, proliferate and initiate self-repair programs after injury, processes that are necessary for restoring vascular homeostasis [18, 19]. Studies elucidating the mechanisms underlying EC recovery and vascular repair after injury are urgently needed to develop novel methods for treating ALI/ARDS.

Vascular endothelial repair depends on the processes of EC proliferation and migration and angiogenesis [20–22]. It is well known that Cdc42 is a member of the Rho GTPase family. We have found that Cdc42 is a key factor participating in several types of cellular biochemical processes, such as cell proliferation, migration and differentiation in different cell types, including epithelial cells, immune cells and tumor cells [23–27]. In addition, in endothelial cells, Cdc42 mediates vascular morphogenesis and maintenance during embryonic development by promoting cell proliferation and migration [26, 28]. We have shown that deleting Cdc42 in ECs throughout the body resulted in embryonic lethality and stillbirth caused by defects in angiogenesis and vasculogenesis [29]. However, it is unknown that whether deleting endothelial Cdc42 has an impact on the vascular endothelial repair and whether Cdc42 could be a regulating role of endothelial recovery after inflammatory injury in ALI/ARDS. Therefore, we proposed a hypothesis that Cdc42 might be a crucial regulator of cell machinery in ECs and thereby promotes endothelial regeneration and vascular recovery following vascular injury in ALI/ARDS.

## Methods

### Mice and genotype identification

The Tie2creER transgenic mice (tamoxifen-treated mice expressing Cre recombinase only on vascular ECs) used in the study were obtained from Yi Zheng’s laboratory at Cincinnati University, USA. The Cdc42^flox/flox^ homozygous transgenic mice used herein were donated by Professor Zheng Yi of the University of Cincinnati, USA. The Td-tomato^flox-stop-flox^ fluorescent indicator mice were also obtained from Yi Zheng’s laboratory. These transgenic mice were maintained in an specific pathogen-free (SPF) animal house and cared for by the Southern Medical University Experimental Animal Center.

Using the Cre/Loxp knockout technique, we crossed the Tie2creER ^+/−^ transgenic mice with Cdc42^flox/flox^ transgenic mice and the td-tomato L-STOP-L mice. After genotyping of the offspring, Tie2creER^+^Cdc42^flox/flox^td-tomato^flox/flox^ mice were selected. After intraperitoneally injection with tamoxifen at a concentration of 5 mg/g for 4 days, endothelial-specific Cdc42 deletion mice were generated (Additional file 1: Figure S1), with auto-red-fluorescence expressed in all of the vascular ECs.

### LPS-induced ALI model and ALI scores

The mice were anaesthetized with 1% pentobarbital (0.1 ml per 20 g i.p.) before receiving LPS intratracheally. The skin was incised to completely expose the trachea, and a micro-needle (50 μl volume) was inserted into the trachea below the cricoid cartilage to deliver LPS (4 μg/kg) at the indicated concentration. The mice were sacrificed at different time points following the procedure. Lung injury severity was assessed according to the guidelines of the American Respiratory Association [30]. The mice at the basal level were treated with tracheal injection of PBS.

### Cell culture and LPS administration

Human pulmonary vascular endothelial cells (HPVECs) (ATCC crl-3244) were grown in ECM (ScienCell, America) containing 5% FBS, 1% P/S and 1% EFGS. After the cells were starved for 12 h in serum-free medium, they were treated with LPS at a concentration of 1 mg/L for 6 h. The medium was then replaced with complete medium, in which the cells were cultured for subsequent experiments.

### Inhibitor treatment and siRNA transfection

After starvation, the cells were treated with a Cdc42-GTP inhibitor (TargetMol, China) at a concentration of 1 mg/L, incubated for 24 h, and prepared for subsequent experiments.

The siRNA-Lipofectamine 2000 complex was prepared for the transfection experiments using siRNA at a concentration of 33 nmol/L. A total of 0.5 ml of the complex was subsequently added to each well of a 6-well plate and supplemented with 1.5 ml of serum-free Opti-MEM, and the plate was then incubated for 6 h at 37 °C. The effect of transfection was tested by WB (Additional file 2: Figure S2). The culture medium was replaced after 48 h, and then the cells were prepared for the follow-up experiments.

### Cell viability and cell proliferation

CCK-8 assay was performed to test cell viability and cell proliferation recovery in cells with or without the Cdc42 gene that were treated with LPS. A total of 1 × 10^4^ cells were seeded in 96-well plates containing complete medium and then incubated with CCK-8 reagent (each 100 μl of medium contained 10 μl of CCK-8 stock solution) for 2 h. The OD value was determined by measuring the absorbance at 520 nm with a microtiter plate reader.

After siRNA transfection, the cells were digested, centrifuged at 1000 rpm for 5 min, and then seeded in 96-well plates for the CCK-8 assay, as described above. The absorbance was directly related to the number of viable or proliferating cells.

### Migration assays

A total of 1 × 10^4^ cells were seeded in 24-well plates and allowed to grow to complete confluence. After starvation, the cells were scratched with a sterile 200-μl pipette tip before being washed with warm medium and photographed at 0, 24 and 48 h after wounding. The migration distance was quantified for subsequent data analysis.

### Tube formation assay

The cells were seeded in 48-well plates pre-coated with a thin layer of Matrigel (BD Biosciences). Culture medium was added to the wells, after which the cells were allowed to form tube-like structures over 24–36 h. After tube formation, CFSE fluorescent dye was used to enhance the visibility of the tubes and networks. Tube formation was quantified as previously described.

### Histology and Immunohistochemical/Immunofluorescence staining

After collection, the lungs were infused with 4% paraformaldehyde (PAF) to inflate all the lobes and maintained in PAF for 24 h before being embedded in paraffin for hematoxylin & eosin (H&E) staining for subsequent blinded histopathologic assessments. The guidelines of the American Respiratory Association were used to assess lung injury severity.

For the immunohistochemical staining experiments, 4-μm tissue sections were deparaffinized and then boiled in antigen retrieval solution (citrate, 3 g; acid, 0.4 g; ddH2O, 1 L; pH 6.0) for 10 min. The sections were subsequently treated with 10% H2O2 for 15 min to block endogenous peroxidase activity before being incubated with the appropriate primary antibodies overnight at 4 °C. A DAB peroxidase substrate kit (Bios, Wuhan, China) was used to visualize the signals.

For the immunofluorescence staining experiments, the lungs were embedded in OCT, cut into 25-μm-thick sections and then treated with blocking buffer (5% BSA with 10% goat BS) for 1 h at room temperature before being incubated with primary antibodies at 4 °C overnight. The sections were subsequently incubated with Alexa488/568-conjugated secondary antibodies and then stained with DAPI for 10 min, after which the fluorescence signals were photographed by microcopy (Leica).

### Assessment of vascular leakage and barrier function in vivo

Mice were injected with Evans Blue dye (20 mg/kg, Sigma) in the tail vein which was allowed to circulate for 3 h before the mice were sacrificed to access pulmonary permeability. Lungs were then collected, and the rights lungs were weighed to determine the ratio of wet-to-dry while the left lungs were homogenized, incubated with formamide (18 h at 60 °C), and centrifuged at 8000 rpm for 30 min. The OD value of the supernatant was determined spectrophotometrically in nm. Extravasated EB concentrations in the lungs were measured by using a standard curve (μg of EB per g of lung tissue) as previously described [31].

### Western blotting

An aliquot of 40 μg of total protein was added to each well of an SDS-PAGE system, which was used to resolve the proteins. The proteins were then transferred to a PVDF membrane (Millipore) and blocked before being incubated with the appropriate primary antibodies overnight at 4 °C. The membranes were subsequently incubated with horseradish peroxidase (HRP)-conjugated goat anti-rabbit IgG (1:3000, 401,353, Millipore) for 1 h at room temperature. Development and fixation were performed using an ECL system (Millipore). The gray levels of each strip were analyzed using Image-Pro plus 6.0 software.

### Statistical analysis

SPSS 22 software was used for statistical analysis. The data are presented as the mean ± standard deviation. Comparisons between two samples were performed with the independent samples t test or the Mann-Whitney U (nonparametric) test, and comparisons of multiple samples were performed using the least significant difference (LSD) method in the presence of homogeneity of variance and Dunnett’s T3 method in the presence of heterogeneity of variance. All statistical analyses were two-tailed tests, and *P* < 0.05 was considered statistically significant.

## Results

### EC-specific deletion of Cdc42 in mice

Deleting Cdc42 in ECs using a Tie2-cre reporter causes embryonic death and prevents exploration of the mature endothelium. To address this problem, we generated an inducible cre-recombination model (Tie2-cre-ER mice) characterized by timed EC-specific deletions of Cdc42 by crossing floxed Cdc42 (Cdc42 ^flox/flox^) mice. In addition, we crossed Td-tomato^flox-stop-flox^ reporter mice with Tie2-cre-ER mice, which enabled us to trace the recombined ECs with a red fluorescence marker. To determine the role of Cdc42 in ECs in adult mice, we generated Cdc42-KO mice (Cdc42^fl/fl^; Tie2-cre-ER). Their littermates served as control mice.

**Fig. 1:**
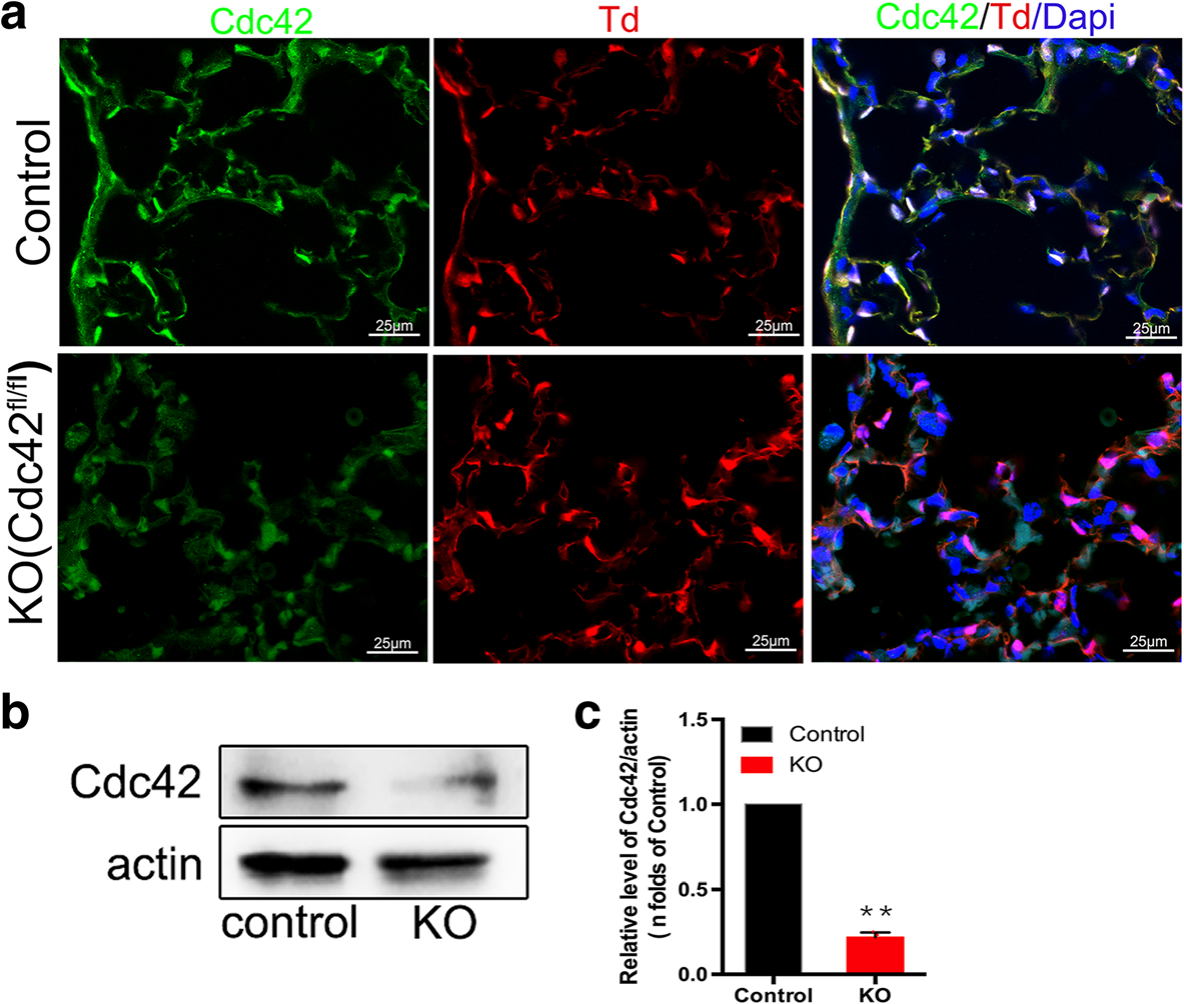
Endothelium-specific knockout of Cdc42 in mice. **a** Fluorescence indicating Cdc42 expression in the lungs. Cdc42 control (Cdc42^fl/+^; Tie2-cre-ER; Td) and Cdc42-KO (Cdc42^fl/fl^; Tie2-cre-ER; Td) mice were pulsed with tamoxifen at 4 weeks after birth and harvested 3 weeks later. The Td-tomato (red) model was used to mark pulmonary vascular ECs. The lung sections were immunostained with antibodies against Cdc42 (green) that distributed within ECs (which appear yellow and exhibit overlapping red staining), as well as other types of cells, in control mice; however, Cdc42 was absent from the endothelium and present in the alveolar epithelium in KO mice, *n* = 4 mice per group. Scale bar, 25 μm. **b** Representative western blotting results showing that Cdc42 expression differed significantly between control and Cdc42-KO mice. The experiment was repeated three times with similar results. **c** Density analysis of the western blotting results. **P < 0.01 (Student’s *t* test)

We then used immunofluorescence staining to confirm the endothelial-specific nature of the Cdc42 deletion (Fig. 1a). The above mentioned red fluorescence marker, which was located specifically on ECs, enabled us to determine whether Cdc42 had been knocked out in ECs. Photographs revealed that Cdc42 was expressed not only on the vascular endothelium but also on the alveolar epithelium and bronchi in control mice. We observed that the green fluorescence indicative of the presence of Cdc42 was absent in vascular endothelial cells in Cdc42-KO mice and was present in other tissues in the lungs of these mice, demonstrating that Cdc42 had been knocked out in pulmonary vascular ECs.

We performed western blotting of lung tissue specimens to confirm the above results and found that Cdc42 protein expression levels were significantly decreased in Cdc42-KO mice compared to control mice (Fig. 1b). Therefore, we were able to undertake additional studies regarding the regulatory effects of Cdc42 on endothelial function and restoration after LPS-induced pulmonary vascular injury.

### Cdc42 depletion impaired inflammation recovery after LPS-induced injury

**Fig. 2:**
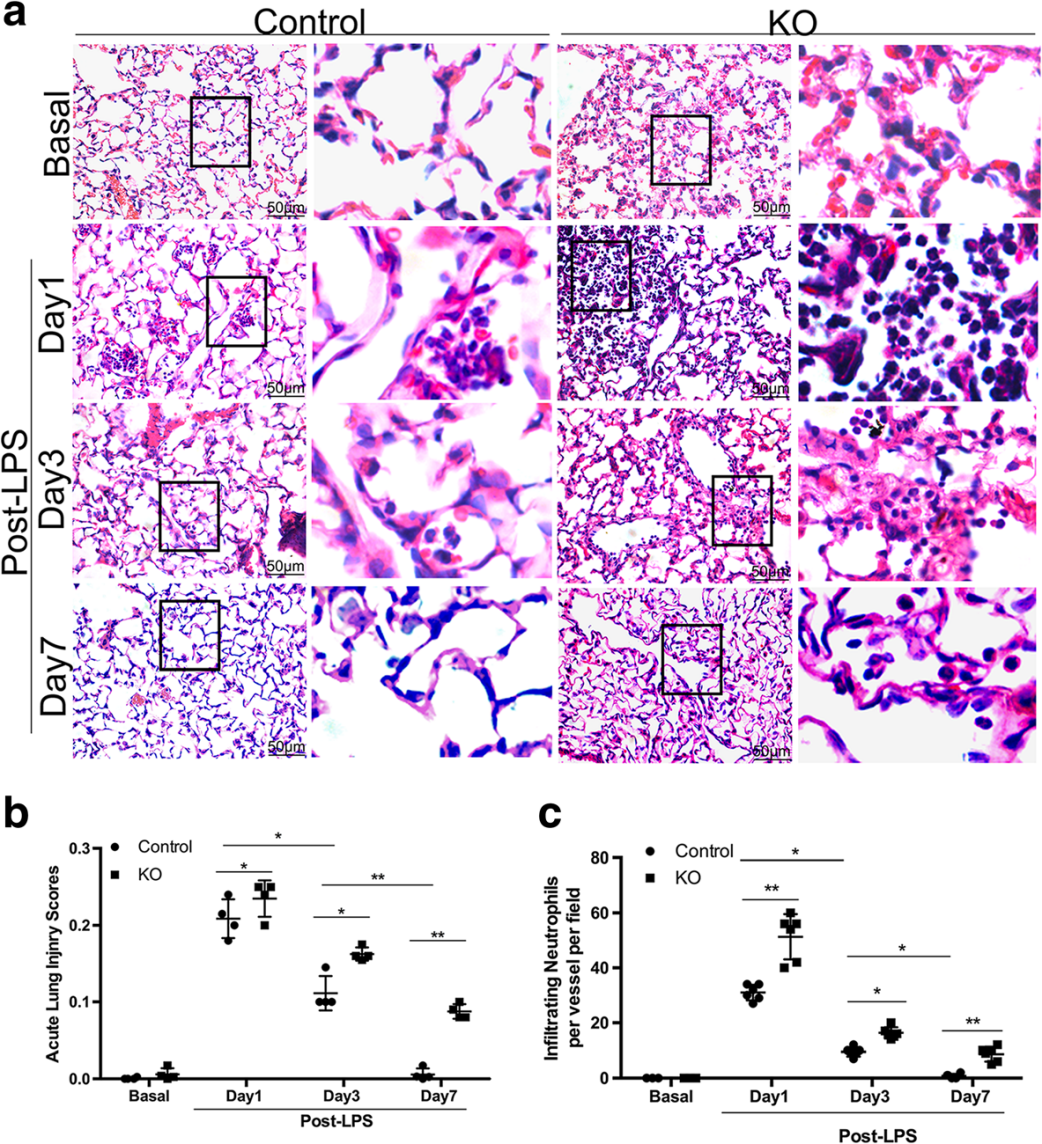
Impaired inflammation resolution in Cdc42-KO mice during the repair phase. **a** H&E staining of lung sections showing inflammatory cell infiltration around vessels and capillaries in control and KO mice, *n* = 4 mice per group. Scale bar, 50 μm. **b** ALI scores at baseline (without LPS) and on days 1, 3 and 7 after LPS-induced injury in control and KO mice, *n* = 4 mice per group. *P < 0.05, **P < 0.01 (Student’s *t* test). **c** Analysis of the number of infiltrating neutrophils per vessels (> 30 μm in diameter), *n* = 6vessels in 4 mice per group. *P < 0.05, **P < 0.01 (Student’s *t* test)

We constructed an LPS-induced ALI mouse model characterized by the absence of the Cdc42 gene in the endothelium to assess the impact of Cdc42 deletions on inflammatory injury progression (Fig. 2a). We scored ALI using a scoring system devised by the American Respiratory Association (Fig. 2b).

ALI scores increased two- or three-fold and peaked at 1 day after LPS-induced injury in the lungs of both Cdc42-KO and control mice (Fig. 2b). These scores decreased significantly on day 3 and returned to baseline on day 7 after LPS-induced injury in the control mice, findings suggestive of a normal recovery. However, the scores for the KO mice were remained higher than the corresponding scores for the control mice on days 3 and 7 post-LPS-induced injury, indicating that the inflammatory injury state persisted in the absence of Cdc42 in these mice.

We then assessed the degree of perivascular inflammatory cell infiltration (Fig. 2c). On day 3 after LPS treatment, the lungs from the Cdc42-KO group still displayed more severe perivascular neutrophil infiltration than the lungs from the control group, which displayed significantly reduced perivascular neutrophil infiltration. In addition, neutrophil uptake was persistently elevated in KO mouse lungs on day 7 after injury, whereas neutrophil uptake had returned to its basal level in control mouse lungs at the same time point, indicating that inflammation resolution was disrupted in the KO group. These results showed that knocking out Cdc42 resulted in increased neutrophil recruitment after LPS challenge and impaired the inflammatory injury recovery process, leading to persistent lung inflammation during the repair phase.

### Cdc42 deficiency disrupted endothelial regeneration and vascular restoration during the repair phase in vivo

We subsequently investigated whether endothelial Cdc42 was required for cell proliferation during the repair process after LPS-induced injury. Proliferating ECs in the lungs were identified based on the expression of the Td-tomato marker, which causes the cells on which it is expressed to appear red, and the BrdU proliferation marker, which causes the cells on which it is expressed to appear green.

**Fig. 3:**
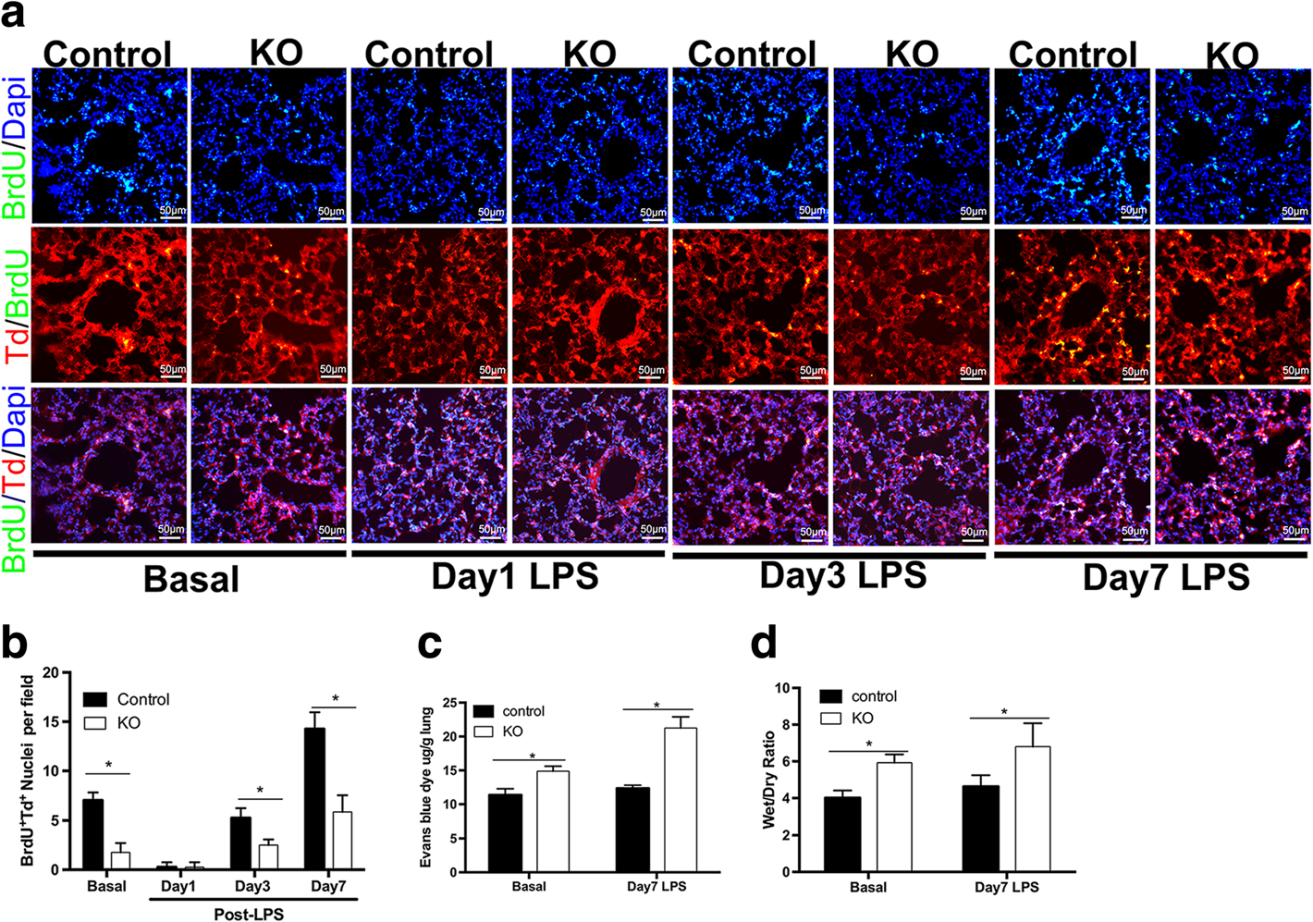
EC proliferation and vascular barrier integrity were impaired in Cdc42-KO mice compared to control mice. **a** Representative micrographs showing EC proliferation. The double-positive cells exhibiting simultaneous green and red fluorescence were proliferating ECs, *n* = 5 mice per group. Scale bar, 50 μm. **b** Graphic presentation of significant differences in cell proliferation, *n* = 5 mice per group. *P < 0.05 (Student’s *t* test). **c** Pulmonary transvascular Evans Blue flux, a finding indicative of defective barrier recovery, in KO mice at baseline and on day 7 during the repair phase, *n* = 4 mice per group. *P < 0.05 (Student’s *t* test). **d** The wet-to-dry weight ratio was increased in Cdc42-KO mice compared to control mice at baseline and on day 7 after LPS challenge, *n* = 4 mice per group. *P < 0.05 (Student’s *t* test)

As shown in Fig. 3a and b, the lungs from the control group exhibited significantly increased cell proliferation during the repair phase after LPS-induced injury compared to that during the recovery phase. We also found that there was a significant difference in cell proliferation between control and Cdc42-KO mice at baseline, suggesting that Cdc42 affects the capacity of ECs to proliferate. In addition, virtually no EC proliferation was noted in KO mice on day 1 after LPS-induced injury, which is similar to the findings in the experiments involving control mice, demonstrating that Cdc42 deficiency inhibited the initiation of vascular recovery after injury. Furthermore, we showed that EC proliferation was significantly decreased in Cdc42-KO mice compared with that in control mice on day 7 after LPS challenge, demonstrating that Cdc42 plays an important role in mediating endothelial regeneration during the repair process.

We then assessed the changes in the vascular recovery function associated with ALI and recovery by measuring the wet weight-to-dry weight ratio and the degree of Evans Blue due extravasation in the lungs on day 7 after lung injury (Fig. 3c, d). We performed the Evans Blue assay to measure vascular permeability to assess the degree of vascular leakage. At baseline, vascular permeability in Cdc42-KO lungs was significantly decreased compared with that in control lungs, indicating that Cdc42 plays a key role in regulating vascular repair. On day 7 of the repair process, the wet-to-dry weight ratio in the control group was close to its basal level; however, the KO group continued to exhibit a high level of vascular leakage, suggesting that the repair process was defective. We showed that Cdc42 deficiency induced a drastic increase in vascular permeability in the corresponding group compared to the control group, which led to ongoing vascular leakage during the repair phase. These findings demonstrated that Cdc42 deficiency impairs vascular integrity and recovery from LPS-induced injury. In addition, our data also showed that the increases in vascular leakage noted on day 7 after LPS challenge occurred concurrently with the increases in perivascular neutrophil infiltration in Cdc42-KO mice during the repair phase.

### Cdc42 is required for EC proliferation and migration and vascular repair in vitro

To determine whether the capacity of the endothelium to regenerate is regulated by Cdc42, we performed siRNA transfection to knock down the Cdc42 protein (siCdc42). Cells treated with scRNA served as control cells. We confirmed that Cdc42 protein expression levels were decreased as a result of successful transfection, as described in Additional file 2: Figure S2. The western blotting results showed that the three RNA fragments had the same effect on transcription. Given that Cdc42 interacts with the active and inactive forms of GTP, we also determined whether GTP-Cdc42 inactivation had negative effects on EC regeneration similar to those of Cdc42 knockdown using a GTP-Cdc42 inhibitor (InhCdc42) in vitro.

**Fig. 4:**
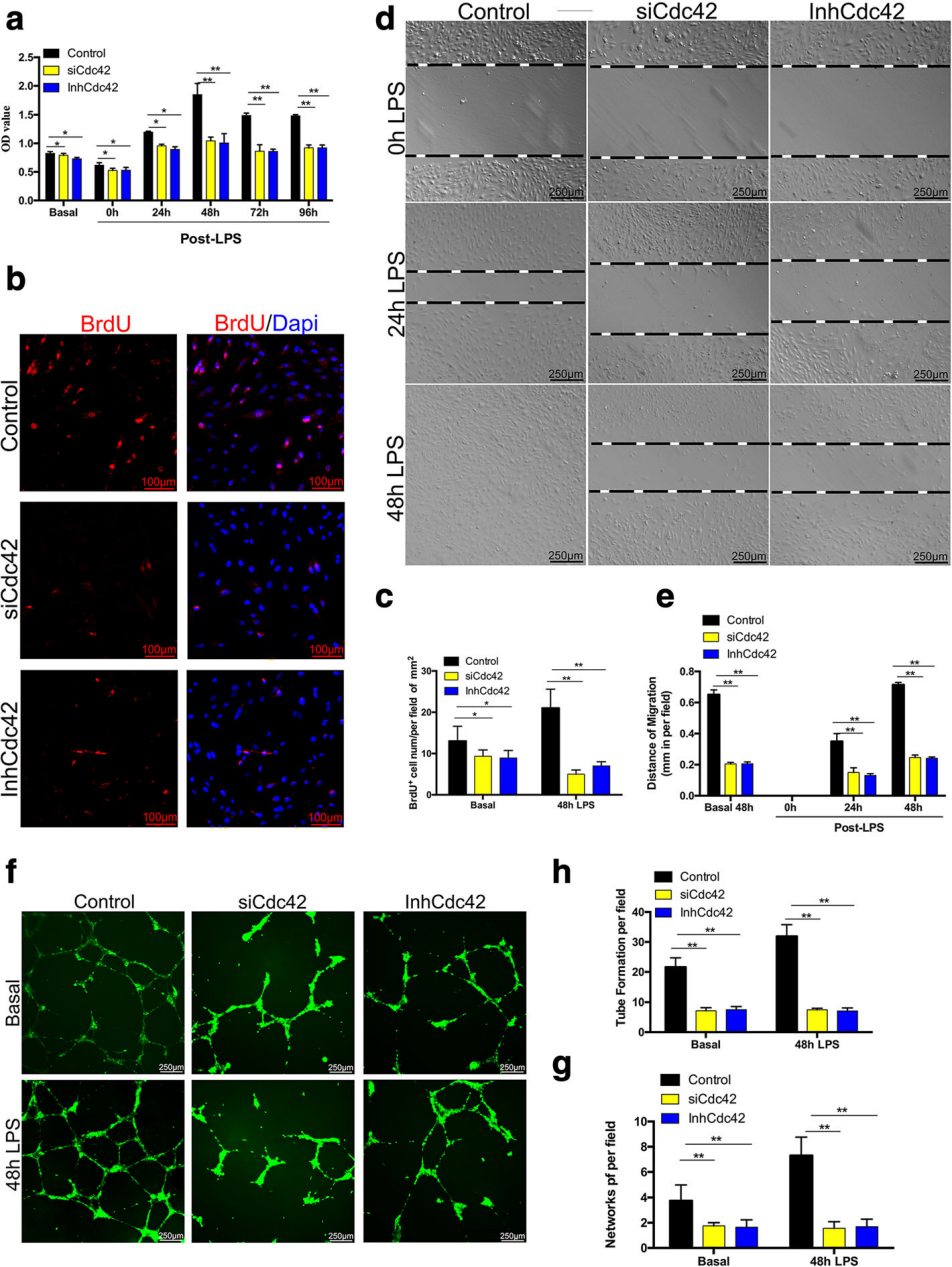
Cdc42 regulates cell proliferation and migration and angiogenesis during the repair process. **a** CCK8 assay showing that cell viability recovery peaked at 48 h after LPS-induced injury in control cells. Representative OD values are shown. In addition, siCdc42 and InhCdc42 cells displayed decreased viability compared to control cells at the same time point. *n* = 4. *P* < 0.05 (Student’s *t* test). **b** Representative micrographs demonstrating the proliferative capacity of ECs in the control, siCdc42 and InhCdc42 groups at 48 h after LPS challenge. The cells were stained with Alexa540-conjugated anti-BrdU antibodies and appear red. The nuclei were stained with DAPI and appear blue, *n* = 4. Scale bar, 100 μm. **c** Graphic presentation of the significant differences in cell proliferation between the groups, *n* = 4. *P < 0.05 **P < 0.01 (Student’s *t* test). **d** Representative micrographs of cell migration at 48 h after LPS challenge, *n* = 4. Scale bar, 250 μm. **e** Graphic presentation of the portion of the scratch line across which the cells had migrated, *n* = 4. **P < 0.01 (Student’s *t* test). **f** Representative micrographs showing vascular repair at baseline (48 h after seeding in the Matrigel-coated wells) and 48 h after LPS challenge. The live cells were stained with CFSE to enhance their visibility (green), *n* = 4. Scale bar, 250 μm (**g**, **h**) Graphic presentation of the numbers of tubes and networks per field, *n* = 4. **P < 0.01 (Student’s *t* test)

We next detected the proliferative, migratory and vascular repair capacities of HPMVECs during the recovery process after LPS-induced injury in vitro. First, we performed CCK-8 assay to test cell viability to determine the normal time required for endothelial recovery after LPS challenge (Fig 4a). At baseline, the siCdc42 and InhCdc42 groups exhibited lower proliferation levels than the control group, indicating that Cdc42 deficiency or inactivation impairs cell viability. The data pertaining to the 24- and 48-h time points after LPS-induced injury showed that cell viability in control cells was significantly increased and peaked at 48 h post-injury, indicating that the recovery of cell viability was initiated in these cells after LPS-induced injury; however, cell viability in siCdc42 and InhCdc42 cells was significantly decreased compared to that in control cells. Cell viability was slightly reduced in the control group at 72 and 96 h after LPS challenge, suggesting that cell viability decreases with time after LPS-induced injury; however, cell viability was significantly decreased in siCdc42 and InhCdc42 cells at the same time points. Therefore, we defined the 48-h time point after LPS-induced injury as the appropriate time point at which we would assess the success of the repair process in subsequent experiments. We then performed the BrdU incorporation assay, which showed that EC proliferation was decreased in siCdc42 and InhCdc42 cells compared to control cells, indicating that Cdc42 deficiency and inactivation impair cell proliferation (Fig. 4b, c). In addition, there was a significant difference in proliferation levels between siCdc42 and InhCdc42 cells and control cells after LPS-induced injury, demonstrating that Cdc42 is required for cell proliferation during the repair phase after LPS challenge.

Cell wound-healing assay showed that the control cells began to migrate at 24 h post-LPS treatment and that wound had healed fully at 48 h after LPS treatment (Fig. 4d, e). The control group migrated significant distances during the 48-h time period, findings consistent with those of the experiments in which post-LPS-induced injury cell viability was assessed. However, we observed significantly slowed migration in the siCdc42 and InhCdc42 cells at 24 and 48 h after LPS-induced injury, demonstrating that Cdc42 deficiency and inactivation inhibits cell migration and healing ability in these cells after LPS challenge. Taken together, these results showed that Cdc42 is a key regulator of cell migration and fusion, which facilitate endothelial restoration, after LPS-induced injury.

EC proliferation and migration are prerequisites for vasculogenesis and angiogenesis. Therefore, we investigated whether Cdc42 influences the angiogenic capacity of HPVECs using a Matrigel-based assay (Fig. 4f, g, h). We assessed angiogenic tube restoration and network formation at 48 h post-LPS-induced injury in cells seeded in a Matrigel-based culture and showed that a significantly smaller number of networks were present in the siCdc42 and InhCdc42 groups than in the control group, showing that Cdc42 supports angiogenesis and that Cdc42 deletion and inactivation disrupt angiogenesis during the repair process. Taken together, these findings demonstrated that Cdc42 plays a role in regulating endothelial regeneration and vascular repair in vitro during the repair phase.

### Cdc42 is an upstream regulator of the PAK1/Akt pathway

**Fig. 5:**
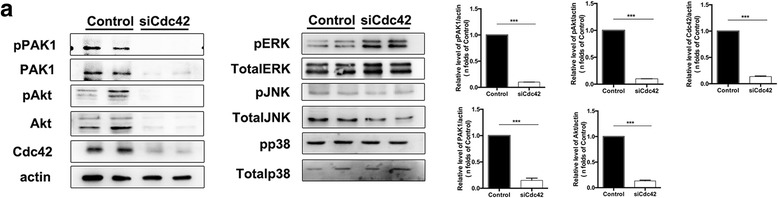
Cdc42 is an upstream regulator of PAK1/Akt signaling. **a** Representative western blotting results showing that PAK1/Akt expression levels were decreased in siCdc42 cells compared to control cells. The experiment was repeated three times with similar results. Density analysis of the western blotting results. **P < 0.01 (Student’s *t* test)

We next observed that siCdc42 caused significant decreases in phosphorylated PAK1 and total PAK1 protein levels in the corresponding group compared to the control group but did not affect ERK, p38, or JNK expression levels (Fig 5). These findings demonstrated that Cdc42 regulates PAK1 expression to mediate endothelial recovery. We also investigated whether the expression of Akt, which is downstream of PAK1, was affected by the presence or absence of Cdc42. We noted significant alterations in phosphorylated and total Akt protein expression. Therefore, we showed that Cdc42 regulates the recovery program through the PAK1/Akt pathway.

## Discussion

In the present work, we investigated the role of endothelial Cdc42 in the regulation of endothelial regeneration and vascular repair after LPS-induced lung injury in vivo and in vitro. We demonstrated that deletion of Cdc42 impairs inflammation resolution, edema alleviation and EC regeneration in mice. We also showed that endothelial Cdc42 knockdown or inactivation disrupts cell proliferation and migration and angiogenesis, thus inhibiting the vascular repair process after inflammation-induced injury in vitro. These results suggest that endothelial Cdc42 plays a key role in preventing defects in vascular inflammatory infiltration resolution and persistent pulmonary vascular leakiness in ALI/ARDS.

We have shown that endothelial Cdc42 plays a role in mediating embryonic survival and development in mice [29]. Because little is known about its regulatory effects during the repair phase after inflammation-induced injury, we focused on the role of Cdc42 in angiogenic repair after LPS-induced injury, as we surmised that this repair process is an important mechanism through which functional gas-exchange interfaces are reestablished in ALI/ARDS.

Using the ALI model established via intratracheal LPS instillation, we showed that deletion of endothelial Cdc42 in mice led to significant exacerbations of perivascular neutrophil infiltration and significant inflammatory cell accumulation in the alveolar cavity following LPS challenge (Fig. 2b, c). In addition, Cdc42 deficiency caused sustained lung inflammation characterized by persistent neutrophil infiltration during the repair phase (on day 7) after LPS challenge, findings consistent with those of our experiments showing that endothelial injury resulted in sustained vascular hyperpermeability (Fig. 3b, c), suggesting that the absence of Cdc42 impairs the restoration of vascular integrity.

The above findings may be explained by the fact that Cdc42 is a fundamental regulator of EC function. We explored the role of Cdc42 in vascular integrity restoration in our study; and then, demonstrated that Cdc42 deficiency has an effect on lung edema and vascular permeability both at baseline and during the repair process after LPS challenge. These data suggested that Cdc42 may represent a novel target through which lung vascular hyper-permeability and edema formation in ALI/ARDS can be regulated.

It has been reported that Cdc42 plays an important role in endothelial cell function and vascular development [7]; thus, we supposed that the above disruptions in inflammation resolution and permeability alleviation that occur in Cdc42-KO mice after LPS challenge may result from vascular ECs regeneration failure in the lungs. Some researchers have observed that cell proliferation peaks at 72 h after LPS-induced injury [20]. The discrepancy between the results of those studies and the results of our study may be attributable to differences in the dose of LPS administered to the mouse model between two the studies. We also investigated the effects of Cdc42 on vascular EC proliferation ability in mice and observed significantly less proliferating cells in Cdc42-KO mice than in control mice both at baseline and on day 7 after LPS challenge, suggesting that ECs fail to proliferate in the absence of Cdc42, a failure that may result in disruptions in vascular barrier functional recovery after LPS-induced injury.

In the mouse experiments, we constructed endothelium-specific Cdc42-KO mice and determined that Cdc42 deletion impaired recovery after LPS challenge. These results indicate that Cdc42 deficiency is a significant cause of defective vascular inflammation resolution and persistent pulmonary vascular leakiness in mice with ALI. And we also explored whether GTP-Cdc42 inactivation had effects on the repair program that were similar to those of Cdc42 knockdown using cell models. Our results indicate that both changes have negative effects on endothelial cell regeneration.

We used siRNA to knock down Cdc42 protein expression and an inhibitor to inactivate GTP-Cdc42 in HPVECs. Previous studies have demonstrated that Cdc42 plays a role in mediating several cellular functions, such cell survival, proliferation, and migration and vascular lumen formation [7, 24, 25, 28, 32]. Our study is the first to explore the role of Cdc42 in the regulation of EC function during the recovery phase after LPS-induced injury, and our results support the hypothesis that the above mentioned impairments in regeneration in HPVECs may be ascribed to the low protein expression of Cdc42 and/or the inactivation of GTP-Cdc42. At 24 and 48 h after LPS injury, the control ECs began to survive, proliferate, migrate and undergo repair, findings indicating that they would likely make a full recovery. In addition, we found that knockdown and inactivation of Cdc42 in ECs inhibits cell proliferation and migration both at baseline and in the repair phase after LPS-induced injury. Moreover, Cdc42 has been found to mediate angiogenesis and vasculogenesis in embryonic development [26]; however, little is known regarding whether Cdc42 regulates vacuole formation during the repair process after LPS challenge. We observed that less networks are present in siCdc42 and InhCdc42 cells than in control cells after LPS injury, suggesting that Cdc42 may play a fundamental role in promoting connections between ECs and vascular networks in repair programs.

Previous studies showed that Cdc42 is a key upstream regulator of PAK1 signaling, which is an important regulator of cell growth, proliferation, and migration [33, 34]. It has been reported that Cdc42-PAK1 complexes regulate cancer cell proliferation [35–37]. In addition, PAK1 also participates in angiogenesis in cancer [38, 39]. Some studies have observed that the PAK1/Akt axis plays the important roles in proliferation in cancer or regulates the process of arrhythmias [40, 41]. PAK1 also plays an important role in vascular remodeling [42], and other studies have shown that PAK1 can activate the canonical MAPK cascade that contributes to cell proliferation [43–45]. Therefore, our study detected PAK1, Akt and MAPK activation in HPMVECs. The siCdc42 cells had significantly lower levels of phosphorylated PAK1 and Akt than control cells, suggesting that Cdc42 functions mainly as an upstream regulator of the PAK1/Akt cascade during the recovery process. We also observed slight increases in phosphorylated ERK levels. These increases likely occur through a negative feedback loop or some other signaling pathway activated by Cdc42 defects and are a phenomenon that warrants further exploration. Thus, our findings indicate that Cdc42 might regulate the PAK1/Akt cascade in pulmonary microvascular ECs to mediate endothelial regeneration and vascular recovery. However, the interaction relationship between PAK1 and the Akt pathway is worthy of further investigations.

In summary, we demonstrate that Cdc42 played an essential role in endothelial cell regeneration and vascular integrity maintenance and might regulate these functions through the PAK1/Akt signaling pathway; thus, Cdc42 could be necessary for repair programs initiated after LPS-induced injury. First, we show that deletion of endothelial Cdc42 in transgenic mice inhibits lung inflammation resolution in ALI models. Then, we demonstrate that Cdc42 knockdown and inactivation inhibits ECs regeneration, thereby disrupting the repair process, providing support for the idea that Cdc42 plays a fundamental role in vascular repair in ALI/ARDS.

## Conclusions

We show that Cdc42 is required for endothelial regeneration and vascular integrity and mediates endothelial recovery after LPS-induced inflammatory injury by activating the PAK1/Akt cascade. Thus, Cdc42 may represent a novel target for therapies designed to treat and prevent ALI/ARDS.

## Additional file

Additional file 1: **Figure S1.** PCR analysis of genomic DNA from the tails of transgenic mice. Cdc42^fl/+^; Tie2-cre-ER; Td represents heterozygous mice without a Cdc42 deletion in the endothelium, while Cdc42^fl/fl^; Tie2-cre-ER; Td represents homozygous mice with an endothelium-specific Cdc42-deletion. The experiment was repeated three times with similar results. (TIFF 395 kb)

Additional file 2: **Figure S2.** The western blotting results showed that the three RNA fragments had the same effect on transcription. The experiment was repeated three times with similar results. (TIFF 172 kb)
