# Supplementary figures and images for: Endothelial Cdc42 deficiency impairs endothelial regeneration and vascular repair after inflammatory vascular injury
Source: Respir Res. 2018 Feb 8;19:27. doi: 10.1186/s12931-018-0729-8 (PMC5806471; doi:10.1186/s12931-018-0729-8)

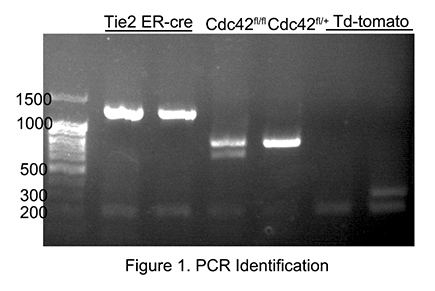

Supplement: Supplementary file 1 — Figure S1. PCR analysis of genomic DNA from the tails of transgenic mice. Cdc42fl/+; Tie2-cre-ER; Td represents heterozygous mice without a Cdc42 deletion in the endothelium, while Cdc42fl/fl; Tie2-cre-ER; Td represents homozygous mice with an endothelium-specific Cdc42-deletion. The experiment was repeated three times with similar results. (TIFF 395 kb) [file 12931_2018_729_MOESM1_ESM.tif]

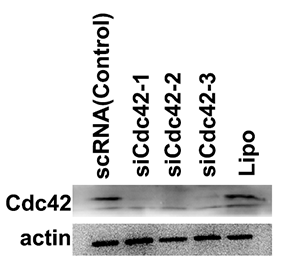

Supplement: Supplementary file 2 — Figure S2. The western blotting results showed that the three RNA fragments had the same effect on transcription. The experiment was repeated three times with similar results. (TIFF 172 kb) [file 12931_2018_729_MOESM2_ESM.tif]
